# Pre2Pub—Tracking the Path From Preprint to Journal Article: Algorithm Development and Validation

**DOI:** 10.2196/34072

**Published:** 2022-04-08

**Authors:** Lisa Langnickel, Daria Podorskaja, Juliane Fluck

**Affiliations:** 1 ZB MED - Information Centre for Life Sciences Cologne Germany; 2 Graduate School Digital Infrastructure in the Life Sciences, Bielefeld Institute for Bioinformatics Infrastructure Faculty of Technology Bielefeld University Bielefeld Germany; 3 Bonn-Aachen International Center for Information Technology University of Bonn Bonn Germany; 4 The Agricultural Faculty University of Bonn Bonn Germany

**Keywords:** preprints, information retrieval, COVID-19, metadata, BERT, Bidirectional Encoder Representations from Transformers

## Abstract

**Background:**

The current COVID-19 crisis underscores the importance of preprints, as they allow for rapid communication of research results without delay in review. To fully integrate this type of publication into library information systems, we developed preview: a publicly available, central search engine for COVID-19–related preprints, which clearly distinguishes this source from peer-reviewed publications. The relationship between the preprint version and its corresponding journal version should be stored as metadata in both versions so that duplicates can be easily identified and information overload for researchers is reduced.

**Objective:**

In this work, we investigated the extent to which the relationship information between preprint and corresponding journal publication is present in the published metadata, how it can be further completed, and how it can be used in preVIEW to identify already republished preprints and filter those duplicates in search results.

**Methods:**

We first analyzed the information content available at the preprint servers themselves and the information that can be retrieved via Crossref. Moreover, we developed the algorithm Pre2Pub to find the corresponding reviewed article for each preprint. We integrated the results of those different resources into our search engine preVIEW, presented the information in the result set overview, and added filter options accordingly.

**Results:**

Preprints have found their place in publication workflows; however, the link from a preprint to its corresponding journal publication is not completely covered in the metadata of the preprint servers or in Crossref. Our algorithm Pre2Pub is able to find approximately 16% more related journal articles with a precision of 99.27%. We also integrate this information in a transparent way within preVIEW so that researchers can use it in their search.

**Conclusions:**

Relationships between the preprint version and its journal version is valuable information that can help researchers finding only previously unknown information in preprints. As long as there is no transparent and complete way to store this relationship in metadata, the Pre2Pub algorithm is a suitable extension to retrieve this information.

## Introduction

The publication of non–peer-reviewed research manuscripts, called preprints, has gained popularity in recent years. Several studies demonstrate the benefits of publishing such preprints; for example, based on the number of citations [1,2]. In addition, the current COVID-19 pandemic shed new light on this type of publication because it allows researchers to communicate new findings quickly, since the publication process is not slowed by peer review.

Although preprints can be a very valuable source of information, especially in times of a pandemic, there is a wide range of quality that cannot be assessed without close examination of the content. The World Health Organization titles the overabundance of both correct and incorrect information during a disease outbreak an “infodemic” [3]. Therefore, preprints must be carefully integrated into existing information infrastructures. Several search portals already include preprints in their database: Europe PMC has been including them for several years [4]; in response to the current health crisis, PubMed launched a pilot project and is integrating COVID-19–related preprints from various preprint servers [5].

In order to ensure rapid central access to COVID-19 preprints and to clearly separate this publication type from reviewed prints, we developed a new preprint service: preVIEW COVID-19 [6,7]. This semantic search engine currently combines more than 40,000 COVID-19–related preprints from 7 different sources. As preprints seem to be established as common first publication type, the problem of content information duplication arises. Consequently, more preprints and journal articles have almost the same content. Especially for articles on COVID-19, where all published information is considered, it is of great importance that as soon as a preprint has been published in a peer-reviewed journal, this information is contained in the metadata and displayed on search portals. Otherwise, this leads to an unnecessary overload for information specialist reading and selecting relevant articles twice. For example, Europe PMC included a filter function where the information is queried from Crossref [8,9]. Crossref is a not-for-profit organization that—besides the allocation of digital object identifiers (DOIs)—offers tools and methods “to help the research community discover, link, cite, and assess scholarly content” [10]. Even though Crossref stores metadata for a total amount of 93,035 journals, books, and conference proceedings [11], Fraser et al [12] recently showed that the information about a corresponding journal article is not completely represented in available metadata. The authors developed an algorithm—based on fuzzy string matching—to automatically query Scopus to find a corresponding journal article.

In this paper, we present the new quality-checked and open source algorithm Pre2Pub, which queries the open access search engine PubMed to find the appropriate journal publications for preprints on the basis of text and author matches. Pre2Pub makes use of recent advances in natural language processing and integrates a Bidirectional Encoder Representations from Transformers (BERT)-based model (sentence-BERT) [13], is evaluated, and is subjected to error analysis. We show that this information is missing in 16% of preprints, making the work of information seekers more difficult, especially during the COVID-19 pandemic. Furthermore, using the semantic search engine preVIEW as an example, this paper presents how the Pre2Pub results can be used as additional information and filtering options in a user-friendly way.

## Methods

### Methods Overview

First, we describe our approach to collect the data from bioRxiv and medRxiv and provide an overview of the generated data sets. Afterward, the developed Pre2Pub algorithm is explained. Finally, we describe the implementation of Pre2Pub into our semantic search engine preVIEW.

### Data

We retrieved data from 2019, 2020, and 2021 from bioRxiv and medRxiv [14]. The servers provide a shared application programming interface (API) for COVID-19–related preprints [15], which is used to distinguish between COVID-19 and non–COVID-19 documents. For further analysis, we stored the following metadata: preprint’s DOI, title, authors list, publication date, published server (bioRxiv or medRxiv), abstract, and (if available) the DOI for the corresponding peer reviewed journal article. Second, we accessed the Crossref API [16] for all retrieved preprints and used the *is-preprint-of* function to determine whether a link to a corresponding peer reviewed version is stored there.

The data consist of a total of 132,339 preprints. Of these, a corresponding journal article link can be found for 51,957 preprints via Crossref and for 62,748 preprints in the metadata of the preprint server. For training and evaluation, we randomly selected 4000 preprints from 2019 and 2020 for which a corresponding journal article is referenced in the preprint server. These were split into training and testing data, with 2000 preprints each. An overview of the retrieved data is provided in Table 1.

**Table 1:** Overview of collected data from bioRxiv and medRxiv from 2019, 2020, and 2021.

Collected data	Preprints, n	Journal digital object identifiers in bioRxiv or medRxiv, n	Journal digital object identifiers in Crossref, n	Distribution (COVID-19/ non–COVID-19), n/n
Total collected data	132,339	62,748	51,957	21,846/110,493
Training data	2000	2000	1433	194/1806
Test data	2000	2000	1452	173/1827

### Pre2Pub Algorithm

**Figure 1:**
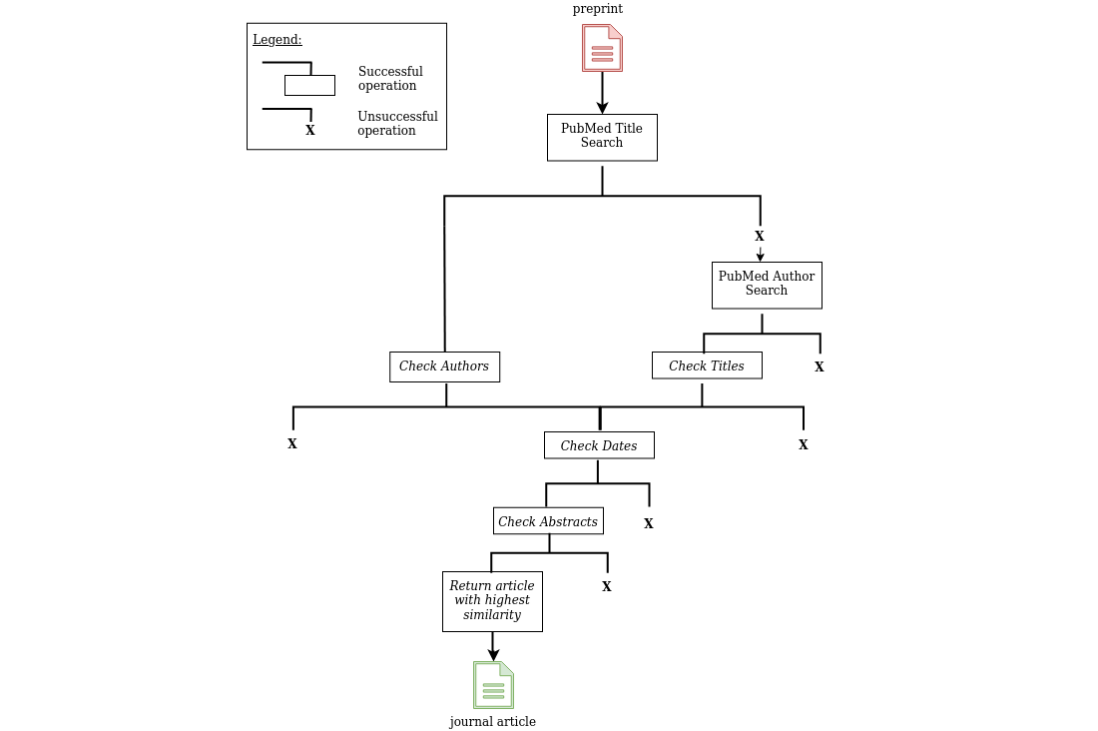
Simplified overview of Pre2Pub’s working mechanism. The input is the metadata of a preprint. Then, by querying the PubMed application programming interface, a title search is performed. If this is successful, the authors are compared by our 5-step method. If not, we perform an author search directly in PubMed and compare then the titles. For the matching articles, the dates are checked and the pairwise abstract similarity is determined. Finally, the article with the highest similarity is returned—if it exceeds the threshold of 0.9.

This algorithm uses a preprint DOI as input and searches for a corresponding journal article in PubMed. We use the E-utilities NCBI service to retrieve PubMed articles on the basis of a defined search query [17]. Pre2Pub consists of 5 steps that are shown in a simplified workflow overview in Figure 1 and described in detail as follows:

1. For all preprints, we used the cleaned title (ie, stop words are removed using the natural language toolkit [18]) as the search term to retrieve a list of PubMed IDs in the *title* field. Retrieval maximum is set to 5 to avoid false-positive results.
2. If the search using the preprint title was successful, the authors of the preprint and the authors of the matched PubMed journal articles are compared. The preprint servers provide the author names in different formats. For example, some servers provide full names with all first names; others provide only last names and first names’ initials, as bioRxiv does. Because PubMed usually stores the complete names (last name, middle name, first name), we queried Crossref as well and extracted the complete preprint’s author names there. To compare the standardized author names between preprint and journal formats, we used the Levenshtein ratio [19] and determined matches using several criteria iteratively developed with the help of the training data: first, we browsed the two lists of authors simultaneously and determine the Levenshtein ratio for each pair. If the value is greater than 0.9, we assume that the authors are identical. If this is not the case, we distinguish the following four cases to consider the authors of the preprints and the authors of the journal article as the same: first, if we find more matched than unmatched author pairs when iterating over the author lists (ie, based on consensus); second, if the first 3 authors of both lists are the same; third, if the first and the last author of preprint and journal article are identical; and fourth, if the first and last authors of the preprint are found in the author list of the journal article (regardless of position).
3. If the title search was not successful, we searched the list of authors in the *author* field. Retrieval maximum was set to 5 in both cases to avoid false-positive results. If successful, we compared the titles of the preprint and the fetched journal articles. To accomplish this, we generated embeddings by making use of Sentence-BERT, “a modification of the pretrained BERT network that use siamese and triplet network structures to derive semantically meaningful sentence embeddings that can be compared using cosine-similarity” [13]. As a pretrained model, we load BioBERT (BioBERT-Base v1.0 [+ PubMed 200K + PMC 270K]) [20]. The threshold is set to 0.95.
4. We compared the dates of publication to ensure that the date of the preprint is older than the date of the peer-reviewed article.
5. We compared the abstract of the preprint to those of the PubMed articles that passed the date check and either the author or title check—depending on which search was successful in the beginning. We generated embeddings and applied the same method as for the title check described in step (3).

Finally, we chose the article with the highest abstract similarity value and, under the condition that it exceeds the threshold of 0.9, linked the found article to the preprint.

### Evaluation Metrics

To evaluate the algorithm, we used the independent test set and determined the precision, recall, and F_1_-score. If our algorithm finds the same DOI, it is considered a true positive; if our algorithm does not yield a matching journal article, we consider it a false negative occurrence; if our algorithm yields another DOI, this is considered both a false positive and a false negative.

### Implementation in preVIEW

As quality differences can exist between a preprint and a peer-reviewed journal article, the availability of a filter option in search portals is important. To display information on whether a preprint is already published in a journal, we integrated Pre2Pub in our semantic search engine preVIEW. Therefore, we updated the information on a weekly basis in the following way: for each preprint, we determined whether a corresponding journal link can be retrieved via the corresponding preprint server (currently only implemented for bioRxiv and medRxiv). If we did not obtain a result, we queried Crossref. If this also failed, we used Pre2Pub. This workflow is applied for all preprints where no information about a corresponding journal article is available. In contrast, if a publication link is already found via the preprint server or Crossref, no update is carried out. For the preprints where either no publication was found previously or the information was only found by Pre2Pub, we checked for updates in the preprint server and Crossref to ensure high quality.

## Results

### Results Overview

This section first provides a general overview of the growth of preprints during the last decade. We then analyzed the collected data set in terms of the number and amount of corresponding journal publications for both preprint servers in the last 2 years. Afterward, we evaluated our developed algorithm on the basis of the independent test set, including error analyses. We further investigated differences in the path from preprint to journal publication for COVID-19–related and non–COVID-19 publications. Finally, we describe the integration into preVIEW’s user interface.

### Above-Average Growth of Preprints in 2020

**Figure 2:**
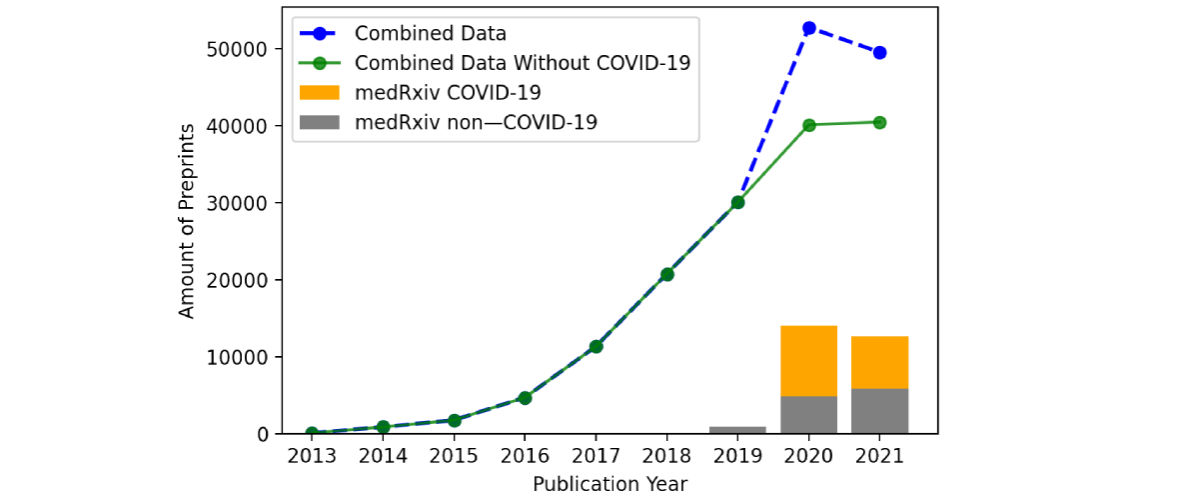
Growth of preprint publications. Combined data include data from both bioRxiv and medRxiv. The blue line comprises all available data, and the green line indicates the amount of preprints minus COVID-19–related papers. Only the medRxiv proportion is depicted as a bar diagram, thereby distinguishing COVID-19 and non–COVID-19 articles.

The COVID-19 crisis led to an increased use of preprint servers because they allow rapid dissemination of research results. The preprint server bioRxiv was launched in 2013 and comprised both biological and medical topics [21]. In the mid-2019, the server medRxiv has been launched additionally to separate these two fields [21]. While only 109 preprints were submitted to bioRxiv in 2013, a total of 30,094 preprints were already submitted to bioRxiv or medRxiv in 2019. As depicted in Figure 2, the publication of COVID-19–related preprints steepens the publication curve and leads to an above-average growth of preprints in 2020.

### Amount of Preprints and Corresponding Journal Articles

**Figure 3:**
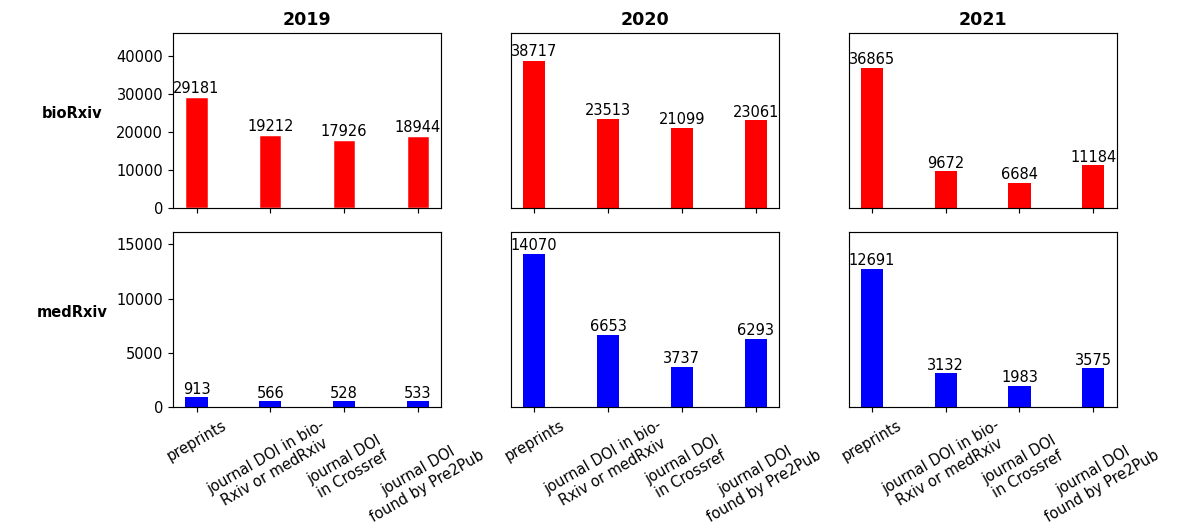
Overview of found journal articles in different sources and through different methods. In the 6 plots, the first bar indicates the total amount of preprints deposited in the preprint servers. Afterward, the absolute amount of found peer-reviewed publications is indicated for all three methods investigated: via the preprint server application programming interface itself, via Crossref, and via Pre2Pub. DOI: digital object identifier.

Significantly more papers are uploaded on bioRxiv than on medRxiv. However, an increase in popularity can be seen in both cases. Whereas only 913 preprints have been posted on medRxiv in 2019, a total of 14,070 preprints were uploaded in 2020. This is partly because medRxiv has been launched in June 2019—before clinical and epidemiological papers were submitted to bioRxiv as well. Figure 3 provides an overview of the retrieved data sets from the preprint servers for the whole years 2019 and 2020.

We independently determined the absolute number of links to a corresponding journal article found by three different methods: (1) the bioRxiv or medRxiv API itself, (2) Crossref, and (3) our algorithm. For every category, the lowest number of articles can be found on Crossref. For 2021, Pre2Pub yielded the most matching publications. For example, for bioRxiv preprints, 9672 corresponding journal articles are referenced in their API. However, Pre2Pub yielded a total of 11,184 matching journal articles.

**Figure 4:**
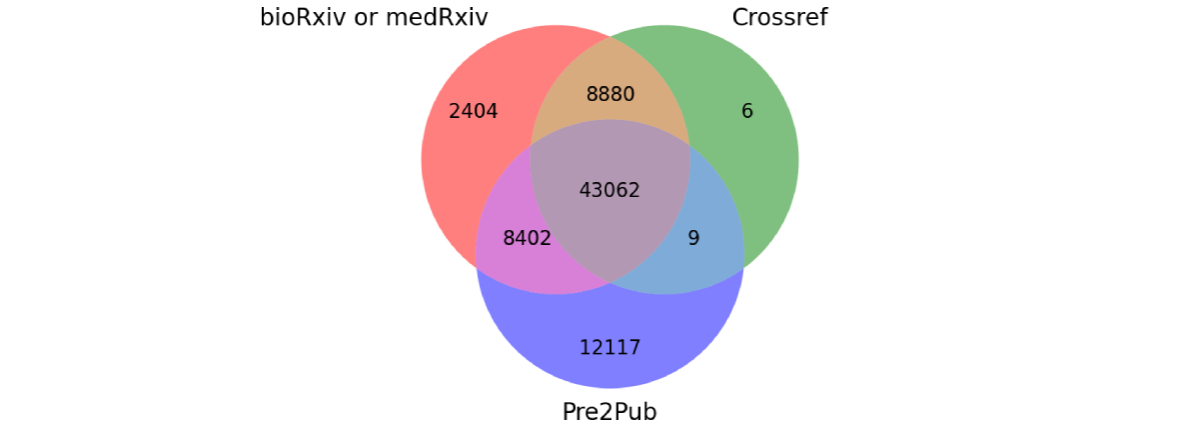
Amount and intersection of found journal articles for 3 different methods. For the combined data set from bioRxiv and medRxiv for 2019, 2020, and 2021, the amount of journal articles is shown for each method. While 43,062 articles were found by all methods, 12,117 articles were only found by Pre2Pub.

While we determined the absolute numbers in the first step, we further investigated the overlap among these three different methods: the Venn diagram in Figure 4 indicates the unique amount that each of the applied methods generates and the respective overlaps, determined on the joint data set (ie, bioRxiv and medRxiv from 2019, 2020, and 2021). Pre2Pub matches a total of 63,590 journal papers—51,473 of which could also be found via the APIs of bioRxiv or medRxiv and Crossref. Hence, 12,117 journal articles were only found by Pre2Pub. This makes up 16.2% of all found journal articles (74,880).

### Evaluation of Pre2Pub

To investigate how reliably Pre2Pub identifies the corresponding journal articles for the preprints, we evaluated Pre2Pub using a test set of 2000 documents; thus, the same document count was used for training. The evaluation results are summarized in Table 2. Similar to training, the evaluation on the independent test set shows promising results with an F_1_-score of 89.29%, resulting from a precision of 99.27% and a recall of 83.29%. On both, the training and the test sets, we achieve a much higher precision than recall; this result is desired because this ensures a higher probability of a false-negative prediction than a false-positive one. The latter would lead to misinformation and therefore needs to be avoided.

An overview of some false-positive and some false-negative matches can be found in Table 3. For some of the false-positive matches, the bioRxiv gold standard was not correct. In the first example, bioRxiv links to an excerpt of conference abstracts and Pre2Pub provides the correct journal article. Similarly, the second example depicts an error on the side of the preprint server. The DOI that is stored in the metadata does not exist—the last 2 digits are missing. In contrast, Pre2Pub finds the correct one.

**Table 2:** Evaluation results of Pre2Pub on training and test data.

Data set	Precision, %	Recall, %	F_1_-score, %
Training data	99.10	82.64	90.13
Test data	99.27	81.14	89.29

**Table 3:** Examples of false-positive and false-negative matches found by Pre2Pub.

Preprint digital objective identifier	Journal digital object identifier found		Error Analysis
	bioRxiv/medRxiv	Pre2Pub	
10.1101/669713	10.1016/j.bpj.2019.11.1890	10.1016/j.bpj.2020.02.011	bioRxiv links only to conference abstracts, whereas Pre2Pub finds the corresponding article
10.1101/845933	10.1016/j.cub.2020.03.005	10.1111/ejn.15056	The correct article is not indexed in PubMed; the found article has the same first and last author and describes a related topic
10.1101/503763	10.1016/j.neuroimage.2019.1161	10.1016/j.neuroimage.2019.116186	Broken link at bioRxiv (incomplete digital object identifier); Pre2Pub finds the correct article
10.1101/549840	10.1128/mBio.00388-19	—^a^	No results via the application programming interface^b^
10.1101/2020.03.06.980631	10.1039/D0GC00903B	—	Article is not indexed in PubMed
10.1101/405597	10.1523/JNEUROSCI.0555-20.2020	—	Titles differ too much

^a^—: not determined.

^b^The search result retrieved from the application programming interface occasionally differing from a manual PubMed search

In the third example, Pre2Pub predicted an incorrect article with a related topic published by the same authors. The correct article is not indexed in PubMed and could therefore not be found. This is one of the major error sources for false negatives since our search is restricted to PubMed; hence, journal articles that are not indexed in PubMed cannot be found. The availability in PubMed has also an influence on false-positive results. If another similar article is found in PubMed with at least partly the same authors, this can lead to a false-positive match. If the correct article is present in PubMed as well, it would be a higher-ranked match; therefore, a false-positive entry would have been omitted.

The last example provides another reason for false negatives: if the titles differ too much, it may happen that the corresponding article is not within the search result, which is restricted to 5 articles.

### Comparison of Preprint to Journal Traversal for COVID-19–Related and Non–COVID-19 Articles

To gain further insight into publication activity before and during the COVID-19 pandemic, we investigated the percentage of preprints that are republished in a journal in 2019, 2020, and 2021. For 2020 and 2021, we differentiated between COVID-19–related and non–COVID-19 articles. While approximately 75% of preprints were republished in a journal in 2019, only a total of 69% of them were published in 2020. Percentage wise, in 2020, more non–COVID-19 preprints than COVID-19–related preprints were published in a journal (69% vs 58%, respectively). However, in 2021, the trend was reversed and approximately 5% more COVID-19–related articles are published in comparison to non–COVID-19 articles. Owing to the large difference in sample amounts when comparing COVID-19 and non–COVID-19 articles, no significant conclusion could be drawn. Moreover, the numbers of published articles are much lower for 2021. This can be explained by the fact that it can take more than a year to finally publish the paper in the journal and the published preprints still undergo review. The results are summarized in Table 4.

**Table 4:** Overview of the amount of bio- and medRxiv preprints that are republished in a journal in 2019, 2020, and 2021.

Year	COVID-19	Amount, n	Published, n (%)
2019	No	30,094	22,776 (75.68%)
2020	No	40,862	28,177 (68.96%)
2020	Yes	11,918	6920 (58.06%)
2021	No	40,441	13,489 (33.35%)
2021	Yes	9024	3518 (38.98%)

### Resulting User Interface Integration

**Figure 5:**
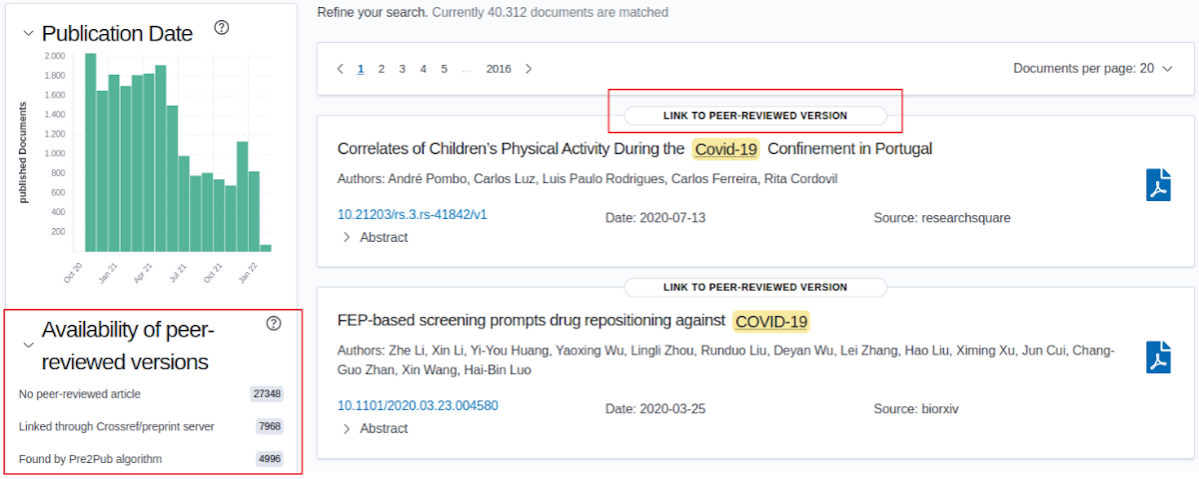
Integration in the search engine preVIEW, publicly available [22]. On the left, an overview of the amount of available peer-reviewed articles can be seen, which can be directly added to the search. On top of each article, if found, the link to the journal publication is provided and opens in a new tab by clicking.

The information about corresponding republished journal versions of the preprint as well as filter functions for search were integrated within our search engine preVIEW. Thus, in our interface, we decided to distinguish between automatically mapped journal links and those provided by the preprint server or Crossref (Figure 5). This yielded three filter options found under the heading “Availability of peer-reviewed version”: (1) no reviewed article, (2) linked through preprint server or Crossref, and (3) found by Pre2Pub algorithm. Importantly, we ran Pre2Pub only for those preprints, where we found a linked journal article neither via the preprint server's API nor via Crossref.

Currently, out of 40,312 preprints, 12,964 have been already published in a journal (approximately 32%). Of them, 7968 corresponding journal publications were found through Crossref or the bioRxiv or medRxiv preprint server. Additional 4996 publications were found by Pre2Pub only—which makes up more than one-third of all found journal articles.

## Discussion

### Principal Findings

In this paper, we first show that preprints have found their place in researchers' publishing behavior, and second that there is currently an information gap when a peer-reviewed version of a preprint appears, making the preprint irrelevant. Third, we provide a solution—the Pre2Pub algorithm—for identifying corresponding journal articles in PubMed and the integration of this information in the preVIEW search engine.

Despite the differences in quality, which may exist between a peer-reviewed and a non–peer-reviewed article, preprints are an appropriate and fast way to publish new results. Preprints have found their place in the publication workflow, as can be clearly seen in Figure 2, which shows an overview of the growth of preprints between 2013 and 2021. This is also true for medical preprints, with a marked increase in medRxiv publications in 2020—the beginning of the COVID-19 pandemic. The main reason for this increase was the high demand for information to quickly gain knowledge to address this crisis.

However, the increasing tendency to publish a preprint first and the peer-reviewed version afterward implies that similar information is published twice, making it difficult to search for relevant information—especially during the COVID-19 pandemic, when preprints have become more relevant. Therefore, it is important to make the information search as transparent as possible and to facilitate the identification and filtering of duplicates.

Consequently, for the publication workflow, it becomes imperative that information about links between publication versions such as preprints and corresponding peer reviewed versions is collected at each replication and is offered by services such as Crossref and DataCite.

During our research, and as also described by Fraser et al [12], we faced the problem that often the information of a journal publication cannot be found in the preprint metadata or via Crossref, although the article can be found in PubMed by a researcher. The underlying reason is that the process is not well established. Often, authors have the option to update the preprint article information to indicate the new journal publication. Some journals also take care of updating this information themselves, ensuring that the preprint is no longer modified. In addition, bioRxiv has its own update process. However, there is neither a uniform solution nor a fixed procedure that an individual researcher must follow.

Until such a process is in place, the presented algorithm Pre2Pub can be used: it searches for the corresponding publication in PubMed to find additional links—in case no results were retrieved via the metadata. Pre2Pub performed robustly as seen in our evaluation. It approached an F_1_-score of 89.92%, resulting from a higher precision than recall (99.27% vs 81.14%, respectively). The significantly higher and near-perfect precision is desirable to minimize the number of false positives. We find more than 12,000 additional publication links for our collected data from the last 3 years (Figure 4). Especially, for 2021, Pre2Pub finds more corresponding journal links, also in absolute numbers. This shows that the process for this type of publication is not well defined and highlights the incompleteness of the metadata provided by the preprint servers.

As a result, duplicated information increases the workload for researchers, which should be avoided, especially in times of a pandemic.

Currently, as shown in our service preVIEW, approximately 32% of COVID-19 preprints are already published in a journal. This result indicates the relevance of such a filtering option in information retrieval to help users of such search portals; for example, information specialists, to find relevant knowledge quickly. As more than one-third of these links were only found by Pre2Pub, it is not enough to simply query Crossref, as is, for example, done by Europe PMC [8].

Next, we plan to extend our algorithm to other literature search portals to enhance our recall further and to integrate the resulting information into the Livivo literature search engine [23].

### Conclusions

Owing to the increased importance of preprints, there is a need to update search engines toward changed information retrieval needs. The need to be able to link a preprint to its corresponding journal article and to filter duplicates was addressed with the retrieval of this information from different resources and the integration within the search frontend of preVIEW.

In the future, we need the commitment of the publishers to request this information from the authors and to store it in the metadata. Until this becomes a reality, services such as Pre2Pub—a freely available algorithm able to find a journal publication for a given preprint—are necessary to retrieve the missing links.

## Abbreviations

API: application programming interface
BERT: Bidirectional Encoder Representations from Transformers
DOI: digital object identifier

## Data and Code Availability

Our code is publicly available on GitHub [25].
